# Infectious complications in pediatric patients undergoing CD19+CD22+ chimeric antigen receptor T-cell therapy for relapsed/refractory B-lymphoblastic leukemia

**DOI:** 10.1007/s10238-024-01339-7

**Published:** 2024-04-25

**Authors:** Xiaochen Wu, Zhanmeng Cao, Zihan Chen, Yi Wang, Hailong He, Peifang Xiao, Shaoyan Hu, Jun Lu, Benshang Li

**Affiliations:** 1 Department of Hematology, Children’s Hospital of Soochow University, Suzhou, 215002 Jiangsu China; 2 Key Laboratory of Pediatric Hematology and Oncology, Department of Hematology and Oncology, Shanghai Children’s Medical Center, Ministry of Health, Shanghai Jiao Tong University School of Medicine, Shanghai, 200127 China

**Keywords:** Infectious complications, CAR-T, Children

## Abstract

Chimeric antigen receptor T-cell (CAR-T) therapy is effective in the treatment of relapsed/refractory acute B-lymphoblastic leukemia (R/R B-ALL); however, patients who receive CAR-T therapy are predisposed to infections, with considerable detrimental effects on long-term survival rates and the quality of life of patients. This study retrospectively analyzed infectious complications in 79 pediatric patients with R/R B-ALL treated with CAR-T cells at our institution. Overall, 53 patients developed 88 infections. Nine patients experienced nine infections during lymphodepletion chemotherapy, 35 experienced 41 infections during the early phase (days 0–+ 30 after infusion), and 29 experienced 38 infections during the late phase (day + 31–+ 90 after infusion). Pathogens were identified in 31 infections, including 23 bacteria, seven viruses, and one fungus. Four patients were admitted to the intensive care unit for infection and one died. In a univariate analysis, there were ten factors associated with infection, including tumor load, lymphodepleting chemotherapy, neutrophil deficiency and lymphocyte reduction, cytokine release syndrome (CRS) and immune effector cell-associated neurotoxicity syndrome (ICANS), etc. In a multivariate analysis, CRS ≥ grade 3 was identified as a risk factor for infection (hazard ratio = 2.41, 95% confidence interval: 1.08–5.36, *P* = 0.031). Therefore, actively reducing the CRS grade may decrease the risk of infection and improve the long-term quality of life of these patients.

## Introduction

Adoptive immunotherapy with chimeric antigen receptor T-cells (CAR-Ts) targeting tumor-specific antigens is a novel treatment for relapsed/refractory acute B-lymphoblastic leukemia (R/R B-ALL). High clinical remission rates have been reported, highlighting its broad application prospects and offering new hope to patients with R/R hematological tumors. However, CAR-T therapy is also associated with life-threatening adverse events, including cytokine release syndrome (CRS), immune effector cell-associated neurotoxicity syndrome (ICANS), hemocytopenia, and infection [1–3]. With the wide application of CAR-Ts in clinics, the management of infections plays a significant role in improving the long-term survival rates and quality of life of patients. According to research in adult patients, the incidence of infectious complications is 20–60%, and many factors directly or indirectly increase the risk [4–6]. At present, few reports exist on CAR-T treatment-related infections in children. Therefore, addressing the gaps in the current understanding of CAR-T therapy-related complications in the pediatric population is an urgent need. In this study, we describe the infectious complications associated with CAR-T therapy in a cohort of pediatric patients and evaluate the potential risk factors for infection.

## Methods

### Patients

This retrospective study was conducted at Children’s Hospital affiliated to Soochow University (Suzhou, China). Seventy-nine children with R/R B-ALL who underwent targeted CD19+CD22+ CAR-T therapy for the first time between September 2018 and January 2022 were included. The patients all achieved remission after receiving treatment, and there was no recurrence within 3 months. Informed consent was obtained from all patients for being included in the study. The study was approved by the Ethics Committee of the Children’s Hospital affiliated to Soochow University and was performed in accordance with the principles of the Declaration of Helsinki.

### Data collection

The clinical data of the patients were collected in three periods (lymphodepletion [LD] chemotherapy and early and late phases), including: (1) general information: sex, age, and history of hematopoietic stem cell transplantation; (2) baseline data before infusion: LD chemotherapy, bone marrow blasts, bone marrow minimal residual disease (MRD), dose of CAR-Ts, and absolute count of neutrophils and lymphocytes in peripheral blood; (3) post-infusion data: peak value of the regulatory T-cell (Treg) proportion (within 1 week after infusion), duration of neutrophil deficiency and lymphopenia in peripheral blood, CRS grade, ICANS grade, use of interleukin (IL)-6 receptor antagonists and glucocorticoids, and admission to the intensive care unit (ICU); and (4) infection-related data: time, symptoms, prognosis, etiology, and medical imaging of the infection and part of the body infected.

### Manufacture of CAR-Ts and LD chemotherapy

CAR-Ts were prepared by the Department of Hematology at the Shanghai Children’s Medical Center (ChiCTR2000032211). The antibody sequence was murine, and the costimulatory molecule was 4-1BB (CD137). The CAR-Ts were cultured for 7 days. The day of CAR-T infusion was defined as d0, and the children underwent LD chemotherapy with cyclophosphamide and fludarabine from d − 4 to − 2. The LD chemotherapy regimen was divided into two groups. In Group A, the total dose of cyclophosphamide was > 1 g/m^2^, and the total dose of fludarabine was > 0.12 g/m^2^. In Group B, the total dose of cyclophosphamide was ≤ 1 g/m^2^, and the total dose of fludarabine was ≤ 0.12 g/m^2^. Baseline data of the two groups had no statistical difference (all *P* > 0.05), and there was comparability between them, Table 1.

**Table 1:** The comparison of baseline data before lymphocyte clearance treatment between the two groups

Variables	Group A (*n* = 68)	Group B (*n* = 11)	*t*/${\chi }^{2}$/*Z*	*P* value
Age [year, M(P25–P75)]	8.5(5.25–11.0)	8.0(4.0–12.0)	0.519	0.604
*Sex*			2.003	0.157
Male (%)	48(70.5)	10(90.9)		
Female (%)	20(29.5)	1(9.1)		
*Prior autologous and/or allogeneic HCT*				0.587*
Yes (%)	6(8.8)	0(0)		
No (%)	62(91.2)	11(100)		

The patients among two groups were diagnosed as R/R B-ALL, all of them only bone marrow relapsed and received CD19+CD22+  CAR-T therapy

*HCT* Hematopoietic stem cell transplantation

*Fisher exact test

## Definitions

### Evaluation standards for adverse reactions

CRS grading refers to Lee’s grading standard [7], and ICANS classification refers to the American Society for Blood and Marrow Transplantation (ASBMT) Consensus Grading classification standard [8].

### Definition of infection

A diagnosis of infection after CAR-T infusion was made according to the clinical symptoms, molecular biology, microbiology, and medical imaging. The onset day of infection was defined as the day on which the diagnostic test was performed. One or more infections may occur in the same patient. Infection in different nonadjacent parts of the body at the same time was considered as an independent event (the lungs, respiratory tract, and paranasal sinuses were considered adjacent) [9], but if the pathogen of the infection was same, it was counted as one infection event. Infection by different pathogens in the same part of the body at the same time was considered as one mixed infection event. Reinfection was defined as the first infection aggravated or reoccurring in the same part of the body during or after treatment. Infections were categorized based on pathogen and site of infection.

### Based on pathogen

#### Bacterial infection

It was divided into bloodstream infections (BSI) and infections in other sites.

1. BSI (including catheter-related) Cultured one or more bacterial from one or more blood samples; if the bacterial isolate is a common bacterium of the skin (such as diphtheria-like bacilli, non-pathogenic mycobacteria, or coagulase-negative staphylococci), and the patient has clinical symptoms, bacteremia is also regarded as an infection.
2. Infections in other sites Patients with compatible symptoms and positive cultures were included.

#### Viral infection

It was divided into respiratory viruses and other viruses.

1. Respiratory virus infection The patients had obvious respiratory symptoms or iconography abnormality and sputum pathogen test (such as nasopharyngeal swab, or alveolar lavage fluid PCR test or blood pathogen antibody, etc.) was positive.
2. Other virus infection The patients had related clinical symptoms, and their blood PCR test (hepatitis B virus, herpes virus, cytomegalovirus, or EB virus) was positive. All patients were screened for virus including AIDS, hepatitis B and C, CMV, EBV and VB19, etc., before the CAR-T therapy, and no infection was found.

#### Fungal infection

Fungal infections were recorded according to the 2008 revised criteria [10].

### Based on site of infection (Table 2)

**Table 2:** Infections were categorized based on site of infection

Site	Definit on
Respiratory tract infection	Symptoms (e.g., fever, cough, or expectoration) and imageology (e.g., chest radiography or chest computed tomography) and/or laboratory tests (sputum culture, alveolar lavage fluid, or viral nucleic acid)
Urinary system infection	Symptoms (e.g., frequent micturition, urgency, dysuria, or hematuria) and laboratory tests (bacteriuria or urine culture or viral nucleic acid)
Digestive tract infection	Symptoms (e.g., diarrhea, abdominal pain, nausea, or vomiting) and laboratory tests (stool routine, stool culture, or viral nucleic acids)

### Infection density

The average number of infections per 100 patient days calculated as the number of infections in different periods (first 30 days and the later 60 days) after transfusion/total number of people days × 100.

### Hypogammaglobulinemia

It was defined as IgG level < 400 mg/dL. All patients received intravenous immunoglobulin (IVIG) replacement every month within 6 months after the therapy (500 mg/kg).

### Treatment of fever and infection

Blood samples were obtained for routine blood tests, C-reactive protein levels, and blood cultures in all patients with fever. Broad-spectrum antibiotics were started empirically, and the antibiotics were adjusted according to the pathogen identification results. Patients with fever after CAR-T infusion and clinical consideration of CRS should be re-evaluated 48 h after fever onset. Antibiotic use should be discontinued in patients with no sign of active infection and negative results on pathogen testing. Antifungal drugs were administered within 90 days of CAR-T therapy to children with previous or current fungal infections. None of the patients received preventive antiviral treatments.

### Statistical analysis

Statistical analysis was performed using SPSS version 24.0 (IBM SPSS Statistics for Windows, Armonk, NY) software. Survival analysis was used to evaluate the clinical factors associated with infections. Independent variables included age, sex, history of hematopoietic stem cell transplantation, LD chemotherapy, CAR-T dose, and CRS grade. The Cox proportional hazard model was used to evaluate the independent risk factors of infection: First, the independent variables were screened by univariate analysis, and variables with *P* < 0.1 in univariate analysis were included in the multivariate model. The Kaplan–Meier curve was used to analyze the correlation between the CRS grade and infection.

## Results

### Clinical characteristics of patients

A total of 79 children were diagnosed as R/R B-ALL and treated with CAR-Ts. The clinical features of the patient cohort, including 58 boys (73%) and 21 girls (27%) with a median age of 8 (5–11) years, are shown in Table 3. Before CAR-Ts therapy, the median number of bone marrow blasts was 6% (range, 3–49%), and the median bone marrow MRD was 9.34 × 10^−3^ leukemia cells (range, 1.0 × 10^−4^–2.47 × 10^−1^ leukemia cells). A total of 55.7% of the patients (*n* = 44) were neutropenic (absolute neutrophil count [ANC] < 0.5 × 10^9^/L), and 55.7% (*n* = 44) presented with lymphopenia (absolute lymphocyte count [ALC] < 0.3 × 10^9^/L). Six (7.6%) children had a history of allogeneic hematopoietic stem cell transplantation. All the patients underwent treatment with fludarabine- and cyclophosphamide-based chemotherapy regimens (Group A, *n* = 11; Group B, *n* = 68). The median infusion of CAR-Ts was 6.8 × 10^6^/kg (range, 5–9.6/kg). In the early phase after CAR-T infusion, the median duration of ANC < 0.5 × 10^9^/L and ALC < 0.3 × 10^9^/L was 13 days (range, 6–31 days) and 6 days (range, 2–11 days), respectively. Thirty-three (41.8%) patients presented with severe CRS (grade ≥ 3) after CAR-T infusion, while severe ICANS (grade ≥ 3) occurred in 9 (11.4%) patients. Fifty-one children (64.6%) were treated with IL-6 receptor antagonists, 33 (41.7%) with glucocorticoids, and 29 (36.7%) with steroids and tocilizumab to treat severe CRS and ICANS.

**Table 3:** Clinical characteristics of the patients

Total	*N* = 79
Age, years (M [P25–P75])	8(5–11)
Sex, male *n* (%)	58(73.4)
CAR-T dose	6.8(5–9.6)
Bone marrow blasts	6(3–49)
Minimal residual disease	9.34 × 10^−3^ (1 × 10^−4^–2.47 × 10^−1^)
Prior autologous and/or allogeneic HCT	6(7.6%)
*Pre-infusion (range)*	
ALC < 0.3 × 10^9^/L	44(55.7%)
ANC < 0.5 × 10^9^/L	44(55.7%)
*Lymphodepleting preparative regimen, n (%)*	
Group A	11(13.9%)
Group B	68(86.1%)
*CRS, n *(%)	
Grade < 3（including grade 0）	46(58.2%)
Grade ≥ 3	33(41.8%)
*ICANS, n* (%)	
Grade < 3（including grade 0）	70(88.6%)
Grade ≥ 3	9(11.4%)
*Use of glucocorticoid*	
Yes	33(41.7%)
No	46(58.3%)
*Use of tocilizumab*	
Yes	51(64.6%)
No	28(35.4%)

*CAR-T* Chimeric antigen receptor T-cell, *HCT* Hematopoietic stem cell transplantation, *ANC* Absolute neutrophil count, *ALC* Absolute lymphocyte count, *CRS* Cytokine release syndrome, *ICANS* Immune effector cell-associated neurotoxicity syndrome

In Group A, the total dose of cyclophosphamide was > 1 g/m^2^, and the total dose of fludarabine was > 0.12 g/m^2^

Group B: the total dose of cyclophosphamide ≤ 1 g/m^2^ and the total dose of fludarabine  ≤ 0.12 g/m^2^

### Infections post-CAR-T therapy

**Fig. 1:**
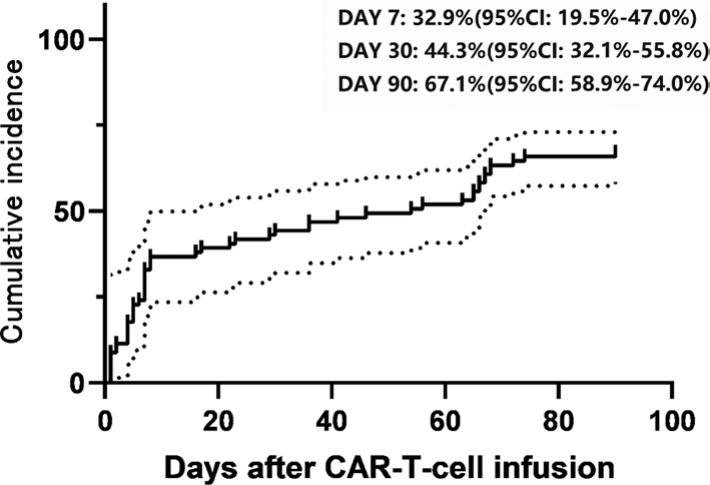
Cumulative incidence of infections post-chimeric antigen receptor T-cell (CAR-T) therapy in pediatric patients

The cumulative incidence of the first infection within 90 days of CAR-T infusion is shown in Fig. 1. The cumulative incidence of the first infection was 32.9% (95% confidence interval CI 19.5–55.8%) by day 7, 44.3% (95% CI 32.1–55.8%) by day 30, and 67.1% (95% CI 58.9–74.0%) by day 90 after CAR-T therapy. The median time to the first infection was 8 days (range, 4–55 days) after infusion, and most infections occurred in the early phase, with an infection density of 1.72. In contrast, infection density was 0.8 in the late phase. Bacterial infections mainly occurred in the early phase (*n* = 20), whereas viral infections were more common in the late phase (*n* = 7) (Table 4).

**Table 4:** Pathogens of infection*

Type of infection	The phase of LD chemotherapy		Days 0–30 post-CAR-T (the early phase)		Days 31–90 post-CAR-T (the late phase)	
	Total episodes	Patients affected	Total episodes	Patients affected	Total episodes	Patients affected
Bacterial infections	0	0	20	16	3	3
Viral infections	0	0	0	0	7	7
Fungal infections	0	0	0	0	1	1

*LD* Lymphodepletion and *CAR-T* Chimeric antigen receptor T-cell

*Pathogens were detected in 31 infections, including 23 bacterial, seven viral, and one fungal infection

In the LD chemotherapy phase, nine patients experienced nine infectious episodes, and no bacterial or viral infections were detected. In the early phase, 41 infections occurred in 35 children, and pathogens were identified in 20 infections, all of which were bacterial. Thirteen children had 14 episodes of bacteremia, of which six were Gram-positive bacteria, eight were Gram-negative bacteria, and all these children had neutropenia. Four children were admitted to the ICU for treatment because of infection, and one died because of grade 4 CRS complicated by *Acinetobacter baumannii* infection of the respiratory tract and *Staphylococcus aureus* bacteremia. In the late phase, 29 children had a total of 38 infectious episodes, and pathogens were detected in 11 cases, including seven viral (herpes virus and human parvovirus detected in plasma by PCR), three bacterial (one Gram-positive bacterium and two Gram-negative bacteria), and one fungal (urinary tract infection caused by *Trichosporon asahii*) infection. Details of the pathogens are presented in Table 5.

**Table 5:** Microbiological description of infection events

Patient number	Phase of LD chemotherapy	Early phase	Late phase
2		*Pseudomonas aeruginosa* (blood)	*Streptococcus pneumoniae* (blood) + EBV (blood)
3		*Stenotrophomonas maltophilia* (urine)	
7		*Staphylococcus warneri* (blood) + *Acinetobacter baumannii* (sputum)	
8		*Staphylococcus* (blood)	
11		*Haemophilus influenzae* (blood) + *S. pneumoniae* (blood) + *Stenotrophomonas maltophilia* (Stool)	CMV (blood)
12			*Escherichia coli* (blood)
13		*Staphylococcus aureus* (blood)	
21		*H. influenzae* (blood)	
30		*Staphylococcus capitis* (blood)	VB19 (blood)
35		*E. coli* (blood)	
42		*Klebsiella pneumoniae* (sputum) + *Staphylococcus epidermidis* (blood)	CMV (blood)
49		*S. pneumoniae* (sputum)	
56		*S. pneumoniae *(blood)	
58			*E. coli* (urine)
59		*Staphylococcus hominis* (blood)	
62			*Trichosporon asahii (*Urine)
63			CMV (blood)
64		*Salmonella* (stool)	
76		*K. pneumoniae* (blood)	
77		*S. hominis* (blood)	VB19 (blood)
79			VB19 (blood)

*EBV* Epstein–Barr virus, *CMV* Cytomegalovirus, and *VB19* Parvovirus B19

### Factors associated with the occurrence of infections

**Fig. 2:**
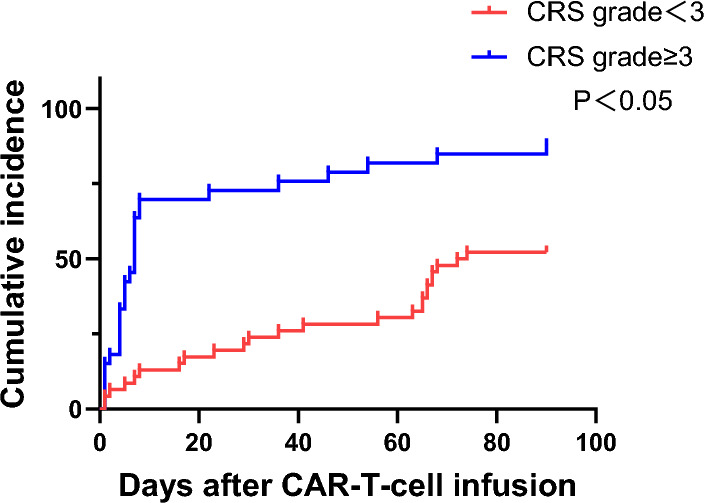
The Kaplan–Meier curve was used to analyze the correlation between cytokine release syndrome (CRS) grades and infection. The infection risk of patients with CRS ≥ 3 after CAR-T infusion increased by 2.41-fold

Univariate and multivariate Cox regression analyses were performed on the clinical factors of the 79 patients, and the risk factors for CAR-T therapy-related infections were assessed (Table 6). Univariate analysis showed that the bone marrow blasts (pre-infusion), MRD of the bone marrow (pre-infusion), lymphopenia (pre-infusion), lymphocyte count before infusion, duration of neutrophil deficiency and lymphocyte reduction after infusion, CRS and ICANS grades, use of IL-6 receptor antagonists and glucocorticoids, ICU admission, and peak value of the Treg proportion (within 1 week after infusion) were associated with the presence of infection (*P* < 0.05). Multivariate analysis showed that CRS ≥ 3 was an independent risk factor for CAR-T therapy-related infection (hazard ratio = 2.41, 95% CI 1.08–5.36, *P* = 0.031) and the infection risk of patients with CRS ≥ 3 after CAR-T infusion increased by 2.41-fold (Fig. 2).

**Table 6:** Association of chimeric antigen receptor T-cell (CAR-T) therapy variables with time to first infection post-CAR-T therapy

Variables	Univariate hazard ratio (95% CI)	*P* value	Multivariate hazard ratio (95% CI)	*P* value
*General information*				
Sex (F vs. M)	1.41(0.79–2.51)	0.242		
Age	1.06(0.98–1.14)	0.114		
*Pre-CAR-T variables*				
Dose of CAR-T cells	0.91(0.83–1.00)	**0.063**	0.98(0.87–1.09)	0.721
Bone marrow blasts	1.01(1.00–1.02)	**0.000**	1.01(0.99–1.02)	0.065
MRD	3.91(1.38–11.07)	**0.010**	0.59(0.10–3.25)	0.545
ANC < 0.5 (Yes vs. No)	1.51(0.86–2.63)	0.147		
ALC < 0.3 (Yes vs. No)	1.99(1.13–3.52)	**0.016**	1.77(0.90–3.47)	0.097
History of HCT (Yes vs. No)	1.66(0.65–4.19)	0.281		
LD chemotherapy (Group A vs. Group B)	2.10(1.05–4.20)	**0.035**	2.01(0.92–4.38)	0.077
*Post-CAR-T variables*				
Duration of ANC < 0.5	1.03(1.01–1.05)	**0.005**	0.99(0.96–1.03)	0.841
Duration of ALC < 0.3	1.03(1.00–1.06)	**0.008**	0.99(0.95–1.02)	0.621
CRS grade (≥ 3 vs. < 3)	3.41(1.95–5.94)	**0.000**	2.41(1.08–5.36)	**0.031**
ICANS grade (≥ 3 vs. < 3)	2.62(1.22–5.64)	**0.013**	1.64(0.66–4.09)	0.285
Use of IL-6 receptor antagonists (yes vs. no)	1.87(1.02–3.42)	**0.040**	1.06(0.50–2.25)	0.874
Use of glucocorticoids (yes vs. no)	2.07(1.20–3.57)	**0.008**	1.05(0.55–2.00)	0.875
Admission to ICU (yes vs. no)	3.63(2.07–6.37)	**0.000**	1.75(0.77–3.98)	0.179
Peak value of Tregs proportion*	1.02(1.00–1.05)	**0.026**	1.00(0.98–1.03)	0.574

Cox proportional hazards model

*M* Male, *F* Female, *CAR-T* Chimeric antigen receptor T-cell, *MRD* Minimal residual disease, *ANC* Absolute neutrophil count, *ALC* Absolute lymphocyte count, *CRS* Cytokine release syndrome, *ICANS* Immune effector cell-associated neurotoxicity syndrome, *HCT* Hematopoietic stem cell transplantation, and *Tregs* Regulatory T-cells

*Peak value of regulatory T-cell proportion within 1 week after CAR-T therapy

The bold numbers: P < 0.1 in univariate analysis and P < 0.05 in multivariate analysis

## Discussion

CAR-T therapy can effectively improve the remission and survival rates of patients with R/R B-ALL. However, adverse events, including CRS, ICANS, infections, and hematological toxicity, are associated with CAR-T therapy. In recent years, CAR-T therapy-related infections have attracted increasing attention. In some prospective clinical trials and retrospective studies, the incidence of infection was approximately 18–60% [11–16]. Our research suggests that the cumulative infection rate within 90 days after CAR-T transfusion was 67.1%, the early infection density was 1.94, and the late infection density was 0.8, which were similar to recent reports [3, 17–19].

Neutropenia is an important risk factor for bacterial infection, and early bacterial infection with CAR-T therapy may be related to multiple neutropenic episodes during this period. Neutropenia is more common after CAR-T therapy, which can be caused by many factors (including CRS and LD chemotherapy) [20–23]. In a clinical study of CAR-T therapy in patients with relapsed/refractory lymphoma, the incidence of neutropenia was 71%, and most of these cases (98%) occurred in the early phase after CAR-T therapy [24]. Fried et al. [22] reported that 72% patients (*n* = 38) with R/R B-ALL had severe neutropenia (grade ≥ 3), and the median occurrence time of neutropenia was by day 17 after the initiation of CAR-T therapy. Our data suggest that 16 children developed infections following CAR-T therapy, with a total of 20 bacterial infections in the early phase. All 16 children developed neutropenia, and seven of these patients had persistent neutropenia (lasting > 20 days). Therefore, we should pay attention to identify bacterial infection, perform anti-infection actively, and avoid cross-infection for patients with neutropenia after CAR-T treatment, especially those with persistent agranulocytosis. In addition, some studies have showed that patients could receive granulocyte–macrophage colony-stimulating factor (GM-CSF) or granulocyte colony-stimulating factor (G-CSF) treatment to decrease the duration of neutropenia, but this approach remains controversial [25–27]. In our center, none of the children was treated with GM-CSF or G-CSF. We plan to perform some researches to evaluate the advantages and disadvantages of this treatment for children who receive the CAR-T therapy in the future.

Viral infections mostly occur in the late phase after CAR-T infusion, which may be related to B-cell aplasia and hypogammaglobulinemia [28]. The “off-target” effect of CAR-Ts (CAR-Ts not only kill malignant B cells, but also target normal B cells) leads to the failure of B-cell regeneration, inducing hypogammaglobulinemia. The incidence of hypogammaglobulinemia varies across treatment centers, with reports indicating an incidence of 20–90% due to differences in research objects, definitions of hypogammaglobulinemia, and methods of immunoglobulin determination [12, 29–31]. In our study, 53 children had complete humoral immunity data including 27 (50.9%) with late-phase hypogammaglobulinemia. Among the 27 children with hypogammaglobulinemia, 11 developed infections, and the pathogens were identified in four children, of which three were viral infections. The infection density in the late phase was lower than that in the early phase, which may be related to the routine monthly infusion of gamma-globulin for patients in our center after CAR-T therapy (until 6 months after treatment), which reduces the incidence of hypogammaglobulinemia. Moreover, lymphocyte and neutrophil counts recovered over time in most cases. In our study, lymphocyte and neutrophil counts recovered in 54 (54/60, 90%) and 48 (48/71, 67.6%) children, respectively, in the late phase. At present, most reports show that respiratory viruses (including influenza virus, parainfluenza virus, metapneumovirus, and respiratory syncytial virus [32]) are the most common pathogens in the late phase of reinfusion, and only a small number of herpes viruses are observed. The reported incidence of cytomegalovirus (CMV) infection is 1–2%, with viremia constituting most cases, whereas organ damage is rare. However, an increasing number of fatal cases of viral infections have been reported in recent years [33, 34]. Respiratory tract infection was the most common in our study; however, the etiological examination of respiratory tract infection has not been checked routinely. Meanwhile, we detected the EBV, CMV, and human parvovirus B19 in the peripheral blood of patients (All patients were screened for virus including AIDS, hepatitis B and C, CMV, EBV and VB19, etc., before the treatment, and no infection was found.) by PCR every month. It showed that CMV and VB19 were common in our data, and all patients were viremia and mainly receive symptomatic treatment.

Fungal infections after CAR-T therapy are rare, with an incidence between 1 and 5% [9, 19], which may be related to persistent neutropenia or the long-term use of glucocorticoids. Our center usually administers antifungal treatment to children with fungal infections during CAR-T treatment. Only one case of urinary tract fungal infection (T. asahii) was detected, and the infection was successfully treated with voriconazole. The child had long-term cytopenia, neutropenia lasting up to 30 days, a history of glucocorticoid use, and persistent application of broad-spectrum antibiotics. These factors increase the risk of fungal infection in children. Therefore, antifungal drugs should be used preventively in children with high-risk factors.

Although infections following CAR-T therapy are common, life-threatening infections are rare. Hill et al. performed a retrospective analysis of 133 patients who underwent CD19 CAR-T therapy, and found that 30 children had 43 infectious episodes, but only two led to death [9]. A report on CAR-T treatment in children and adolescents showed that two of 39 patients died. One patient died of rhinocerebral mucormycosis and E. faecalis disseminated infection, and the other patient died of polymicrobial bloodstream infection with E. faecalis and *S. epidermidis* [3]. In this study, only one child died of grade 4 CRS complicated by infection, which was caused by *A. baumannii* infection in the respiratory tract and *Staphylococcus wallichii* bacteremia. Following treatment with imipenem, amikacin, and voriconazole, the oxygen saturation and blood pressure could not be maintained at stable levels, and the patient died of multiple organ dysfunction syndrome and septic shock. Early identification of infection and active anti-infection therapy are one of the effective strategies to reduce the mortality of CAR-T therapy-related infection. Therefore, when children have symptoms of infection, we should promptly identify the infection site, conduct pathogen identification, actively and empirically use broad-spectrum antibiotics, regularly evaluate the severity of infection, and adjust antibiotics according to the pathogen test results.

We also analyzed the clinical factors related to infection and found that they were related to tumor load, lymphodepleting chemotherapy, neutrophil deficiency and lymphocyte reduction, CRS and ICANS, etc. High tumor load and intensive lymphocyte clearance usually lead to an increased incidence of severe CRS and ICANS [35, 36], and patients with severe CRS have a higher risk of infection [3, 9]. CRS is one of the most common adverse reactions after CAR-T therapy and typically occurs 1–14 days after CAR-T infusion for a duration of approximately 1–10 days, with an incidence of 30–100% while the incidence of CRS grade ≥ 3 being approximately 10–30% [11]. CRS is mainly characterized by fever, hypotension, decreased pulse oxygen, and toxicity of various organs [32]. These symptoms are often difficult to distinguish from sepsis caused by bacterial infection, so we may inevitably overestimate the incidence of infection in the early phase. Patients with severe CRS often require admission to the ICU. Indwelling catheters (central venous catheters, urinary catheters, and tracheal catheters) in the ICU increase the risk of infection, and patients often require glucocorticoid and/or IL-6 receptor antagonist treatment, which may inhibit the ability of the patient’s immune system to respond effectively to pathogens [28]. A single-center study on rheumatoid arthritis showed that the use of IL-6 receptor antagonists was associated with an increase in infection [37]. However, Frigault et al. noted that IL-6 receptor antagonist use in patients after CAR-T treatment was not associated with the occurrence of infection [38]. Regarding the association between glucocorticoid use and infection risk, current findings have yielded conflicting results with some studies showing no increased infection risk [17] and others demonstrating an increased infection risk [39]. These contradictory results may be due to the selection bias of different studies as different research centers have different standards for the selection of research participants, definition of infection, and use of antibacterial drugs. In summary, owing to the comprehensive effects of many factors, severe CRS increases the risk of infection in patients.

Interestingly, we also found that Tregs were related to infections after CAR-T therapy, and the risk of infection increased with an increase in the peak value of the Treg proportion. Tregs are a subset of T cells with strong negative immunoregulatory functions that actively inhibit the activation, amplification, and function of other immune cells, thus regulating the intensity and duration of the immune response and maintaining immune homeostasis in vivo [40–42]. Sustained high expression of Tregs inhibits the activation and expansion of tumor antigen-specific effector T cells, affecting the curative effect of CAR-Ts, further aggravating the immune deficiency of patients, and increasing the risk of infection. Therefore, inhibiting the activation of Tregs, when necessary, may promote the tumor-killing effect of CAR-Ts and reduce the risk of infection. We intend to explore this intriguing aspect in our future clinical work.

After the remission of CAR-T therapy in our center, most children underwent hematopoietic stem cell transplantation within approximately 90 days, and long-term infection after CAR-T transfusion could not be tracked. Further research is required to generate robust data on etiology and immunology to address this limitation.

The incidence, pathogens, and severity of infection after CAR-T therapy are affected by many factors. Therefore, integrating the experience of CAR-T therapy for various hematological diseases is important to better understand related infectious complications and formulate the optimal infection management strategy to improve the safety and effectiveness of CAR-T therapy for children with R/R B-ALL.
